# Supplementary material for: Audiovisual adaptation is expressed in spatial and decisional codes
Source: Nat Commun. 2022 Jul 7;13:3924. doi: 10.1038/s41467-022-31549-0 (PMC9262908; doi:10.1038/s41467-022-31549-0)
Supplement: Supplementary file 2 — Reporting Summary [file 41467_2022_31549_MOESM2_ESM.pdf]

nature research

Corresponding author(s): Máté Aller

Last updated by author(s): Mar 31, 2022

Reporting Summary

Nature Research wishes to improve the reproducibility of the work that we publish. This form provides structure for consistency and transparency in reporting. For further information on Nature Research policies, see our Editorial Policies and the Editorial Policy Checklist.

Statistics

For all statistical analyses, confirm that the following items are present in the figure legend, table legend, main text, or Methods section.

n/a	Confirmed	
☐	☒	The exact sample size (n) for each experimental group/condition, given as a discrete number and unit of measurement
☐	☒	A statement on whether measurements were taken from distinct samples or whether the same sample was measured repeatedly
☐	☒	The statistical test(s) used AND whether they are one- or two-sided<br>*Only common tests should be described solely by name; describe more complex techniques in the Methods section.*
☒	☐	A description of all covariates tested
☐	☒	A description of any assumptions or corrections, such as tests of normality and adjustment for multiple comparisons
☐	☒	A full description of the statistical parameters including central tendency (e.g. means) or other basic estimates (e.g. regression coefficient) AND variation (e.g. standard deviation) or associated estimates of uncertainty (e.g. confidence intervals)
☐	☒	For null hypothesis testing, the test statistic (e.g. F, t, r) with confidence intervals, effect sizes, degrees of freedom and P value noted<br>*Give P values as exact values whenever suitable.*
☐	☒	For Bayesian analysis, information on the choice of priors and Markov chain Monte Carlo settings
☐	☒	For hierarchical and complex designs, identification of the appropriate level for tests and full reporting of outcomes
☐	☒	Estimates of effect sizes (e.g. Cohen's d, Pearson's r), indicating how they were calculated

Our web collection on statistics for biologists contains articles on many of the points above.

Software and code

Policy information about availability of computer code

Data collection	Audiovisual stimuli were presented and behavioral responses were collected with Psychtoolbox v3.0.11 (http://psychtoolbox.org/) running under MATLAB R2011b (MathWorks Inc.).<br>EEG Data was recoded with BrainVision recorder Professional 1.20.0601 (Brain Products).<br>Eye movement data was collected with the EyeLink 1000 system running Eyelink II CL v5.09 software (SR Research).
Data analysis	Behavioural data were analysed with MATLAB R2018b (MathWorks Inc.) using custom code.<br>Psychometric function fits were performed using Palamedes toolbox v1.7.0 (www.palamedestoolbox.org)<br>fMRI data was pre-processed using SPM12 v7219 (http://www.fil.ion.ucl.ac.uk/spm/);<br>EEG pre-processing was done using FieldTrip version 2f8ec8892 (https://www.fieldtriptoolbox.org/)<br>Multivariate decoding on fMRI and EEG data were done using LIBSVM 3.20 (https://www.csie.ntu.edu.tw/~cjlin/libsvm/)<br>Pattern component analyses on fMRI and EEG data were performed using pcm_toolbox version 6c7a218 (https://github.com/jdiedrichsen/pcm_toolbox). Visualization of representational dissimilarity matrices was performed using rsatoolbox version 445e8c6 (https://github.com/rsagroup/rsatoolbox_matlab).<br><br>Code used in the manuscript can be accessed here: https://github.com/allermat/audiovisual_adaptation_fMRI_EEG

For manuscripts utilizing custom algorithms or software that are central to the research but not yet described in published literature, software must be made available to editors and reviewers. We strongly encourage code deposition in a community repository (e.g. GitHub). See the Nature Research guidelines for submitting code & software for further information.

Data

Policy information about availability of data

All manuscripts must include a data availability statement. This statement should provide the following information, where applicable:

- Accession codes, unique identifiers, or web links for publicly available datasets
- A list of figures that have associated raw data
- A description of any restrictions on data availability

Source data are provided with this paper. The processed data files necessary to reproduce the results using the shared analysis code are available at: https://doi.org/10.6084/m9.figshare.19469861.v2. The raw data are available to other researchers upon request, because of constraints imposed by the ethics approval under which this study was conducted.

Field-specific reporting

Please select the one below that is the best fit for your research. If you are not sure, read the appropriate sections before making your selection.

☐ Life sciences ☒ Behavioural & social sciences ☐ Ecological, evolutionary & environmental sciences

For a reference copy of the document with all sections, see nature.com/documents/nr-reporting-summary-flat.pdf

Behavioural & social sciences study design

All studies must disclose on these points even when the disclosure is negative.

Study description	The study is an experimental quantitative study on healthy human participants, collecting behavioral, fMRI, and EEG data.
Research sample	15 participants (10 females, mean age = 22.1; SD = 4.1) participated in the psychophysics study. Five of those participants (4 females, mean age = 22.2; SD = 5.1, one author of the study, A.M.) completed the fMRI and EEG experiments. The sample was recruited among the students of the University of Birmingham, Birmingham, UK using email announcement and the Sona Research Management system. We strove to accommodate a broader age range and educational background, yet the sample is not representative of the whole population.<br><br>A sample size calculation using GPower determined sufficient power (>0.95) using a sample size of 15 (for clear behavioral effects of the experimental paradigm). Having established a robust recalibration effect at the behavioural level across participants, our aim was to maximize the reliability of the within participant estimates of the EEG and fMRI. Therefore, we included four EEG and four fMRI measurement days for each participants. Following previous studies (Rohe & Noppeney, 2015; Smith & Little, 2018), we selected the six participants from the psychophysics part of the study with the highest recalibration effect size to participate in the fMRI and EEG parts of the experiment.
Sampling strategy	The sampling procedure was to include participants meeting the inclusion/exclusion criteria by means of response to recruitment emails and participation in a pre-screening session, until the desired sample size was reached.
Data collection	Visual and auditory stimuli were presented using Psychtoolbox version 3.0.11 under MATLAB R2011b (MathWorks Inc.) on a MacBook Pro running Mac OSX 10.6.8 (Apple Inc.). In the psychophysics and EEG experiments, participants were seated at a desk with their head rested on a chinrest. Two accessory rods were mounted on the chin rest serving as forehead rest and allowing stable and reliable head positioning. Visual stimuli were presented at a viewing distance of 60 cm via a gamma-corrected 24'' LCD monitor (ProLite B2483HS, iiyama Corp.) with a resolution of 1920 x 1080 pixels at a frame rate of 60 Hz. Auditory stimuli were delivered via circumaural headphones (HD 280 Pro, Sennheiser electronic GmbH & Co. KG) in the psychophysics experiment and via in-ear earphones (E-A-RTONE GOLD, 3M Company Auditory Systems) in the EEG experiment. Participants used a standard USB keyboard for responding. In the fMRI experiment, visual stimuli were back projected to a plexiglass screen using a D-ILA projector (DLA-SX21, JVC, JVCKENWOOD UK Ltd.) with a resolution of 1400 x 1050 pixels at a frame rate of 60 Hz. The screen was visible to the subject through a mirror mounted on the magnetic resonance (MR) head coil and the eye-to-screen distance was 68 cm. Auditory stimuli were delivered via a pair of MR compatible headphones (MR Confon HP-VS03, Cambridge Research Systems Ltd). Participants responded using a two-button MR-compatible keypad (LXPAD 1x5-10M, NATA Technologies). During the psychophysics and EEG experiments the researcher (M.A.) was the only person present during data collection. During the fMRI data collection two researchers (A.M. and M.A.) were present. In all cases the researchers were not blinded to the study hypothesis, but they were blinded w.r.t. to the experimental condition which participants were presented with because they were outside the testing room/fMRI scanner during the experiment.
Timing	Experimental data collection started on 15th November 2016 and finished on 4th June 2017
Data exclusions	One participant was excluded from the fMRI experiment after 3 recording days because the behavioural performance did not meet all a priori defined criteria (sensitivity index d' during the adaptation phase was ≤ 2.5).
Non-participation	No participants dropped out/declined participation.
Randomization	Participants were not allocated in different groups

Reporting for specific materials, systems and methods

We require information from authors about some types of materials, experimental systems and methods used in many studies. Here, indicate whether each material, system or method listed is relevant to your study. If you are not sure if a list item applies to your research, read the appropriate section before selecting a response.

Materials & experimental systems

n/a	Involved in the study
x	Antibodies
x	Eukaryotic cell lines
x	Palaeontology and archaeology
x	Animals and other organisms
	x Human research participants
x	Clinical data
x	Dual use research of concern

Methods

n/a	Involved in the study
x	ChIP-seq
x	Flow cytometry
	x MRI-based neuroimaging

Human research participants

Policy information about studies involving human research participants

Population characteristics	The sample was recruited among the students of the University of Birmingham, Birmingham, UK using email announcement and the Sona Research Management system. We strove to accommodate a broader age range and educational background, yet the sample is not representative of the whole population.
Recruitment	Participants for the psychophysics part of the study were recruited among the students of the University of Birmingham, Birmingham, UK using email announcement and the Sona Research Management system. One participant of the study was also an author (A.M.). Following previous studies (Rohe & Noppeney, 2015; Smith & Little, 2018), we selected the six participants from the psychophysics part of the study with the highest recalibration effect size to participate in the fMRI and EEG parts of the experiment. Participants were compensated with £6 per hour for behavioural and £8 per hour for fMRI and EEG sessions.
Ethics oversight	Research ethics committee of the University of Birmingham (approval number: ERN_11_0470AP4)

Note that full information on the approval of the study protocol must also be provided in the manuscript.

Magnetic resonance imaging

Experimental design

Design type	Task fMRI with mixed event/block design
Design specifications	The fMRI design included three phases: (i) unisensory auditory pre-adaptation, (ii) audiovisual adaptation (AV- or VA-adaptation) and (iii) unisensory auditory post-adaptation (postAV-adaptation or postVA-adaptation). In the pre- and post-adaptation phases, the auditory stimuli were presented in a pseudorandomized order in ~36 s blocks of 18 trials interleaved with 6 s fixation (stimulus onset asynchrony (SOA) = 2000 ms ± 200 ms jitter). In the audiovisual adaptation phase, the audiovisual stimulus pairs were presented in ~3 min blocks of 360 trials (SOA = 500 ms). In the pre-adaptation phase, one run consisted of 10 blocks (i.e., 18 trials x 10 blocks = 180 trials) and 9 fixation intervals and lasted for ~7 minutes. After 5 blocks we inserted a longer fixation interval of 15 s. In the audiovisual adaptation and post-adaptation phases, one run included 2 audiovisual adaptation and 10 post-adaptation blocks and lasted for ~14 minutes. Each run started with an audiovisual adaptation block followed by 5 post-adaptation blocks then repeating this pattern again. After the audivisual adaptation block or 5 post-adaptation blocks we inserted a fixation of 15 s.<br>In total, the pre-adaptation phase included 3600 auditory trials (i.e., 18 trials per block x 10 blocks per run x 5 runs per day x 4 days); the audiovisual adaptation phase included 5760 audiovisual adaptation trials for the (left) VA-adaptation and the same number of trials for the (right) VA-adaptation (i.e., 360 trials per block x 2 blocks per run x 4 runs per day x 2 days); the post-adaptation phase included 1440 auditory trials for postAV-adaptation and the same number of trials for postVA-adaptation (i.e., 18 trials per block x 10 blocks per run x 4 runs per day x 2 days).
Behavioral performance measures	Participants responded only on a fraction of 'response trials', i.e., 22% in auditory pre- and post-adaptation and 10% in AV- and VA-adaptation.<br>Behavioural performance was assessed based on the button presses by means of the signal sensitivity index d' (d'= Z (p_hit)- Z(p_false_alarm), where Z refers to Z-score and p_hit and p_false_alarm are the hit and false alarm rates, respectively).<br>Each participant was required to meet a priori defined criteria of sensitivity index d' > 2.5 in pre-, and post-adaptation as well as adaptation phases. We report across participants' mean +/- SEM d' values .

Acquisition

Imaging type(s)	Structural, functional
Field strength	3T
Sequence & imaging parameters	Structural: T1-weighted anatomical images (TR/TE/TI, 7.4/3.5/min. 989 ms; 176 slices; image matrix, 256 x 256; spatial resolution, 1 x 1 x 1 mm3 voxels) .<br>Functional: T2*-weighted echo-planar images (EPI) with blood oxygenation level-dependent (BOLD) contrast (fast field echo; TR/TE, 2800/40 ms; 38 axial slices acquired in ascending direction; image matrix, 76 x 75; slice thickness, 2.5 mm; interslice gap, 0.5 mm; spatial resolution, 3 x 3 x 3 mm3 voxels). A total of 8016 scans were acquired per participant.
Area of acquisition	Whole brain

Diffusion MRI ☐ Used ☒ Not used

Preprocessing

Preprocessing software	The data were preprocessed with Statistical Parametric Mapping (SPM12; http://www.fil.ion.ucl.ac.uk/spm/). The fMRI scans from each participant were realigned using the first as a reference, unwarped and slice-time corrected. The time series in each voxel was high-pass filtered to 1/128 Hz. The fMRI images were spatially smoothed with a Gaussian kernel of 3 mm FWHM.
Normalization	The fMRI images were analysed in native space, data were not normalized.
Normalization template	The fMRI images were analysed in native space, data were not normalized.
Noise and artifact removal	Motion parameters were calculated during realignment and included as nuisance covariates in the data analysis.
Volume censoring	No volume censoring was applied.

Statistical modeling & inference

Model type and settings	At the second, between-subjects level we performed statistical inference on the regional mean BOLD response and finescale BOLD-response patterns of 5 ROIs.<br>At the first, within-subject level, the data were modelled in a mixed event/block design. In the unisensory auditory pre- and post-adaptation phases, unisensory sound stimuli were modelled as events separately for each of our 7 (sound location) x 2 (response vs. non-response) x 3 (pre, post-VA, post-AV) conditions. In the adaptation phases, audivisual stimuli were modelled as blocks separately for the 3 (visual locations) x 2 (VA vs. AV adaptation) conditions. Condition-specific effects for each subject were estimated according to a general linear model (GLM). To minimise confounds of motor response, we limited all subsequent fMRI analyses to the parameter estimates pertaining to the 'non-response' trials.<br>For the mean region BOLD-response analysis, we computed contrast images comparing auditory stimulus at a particular location > fixation in each subject resulting in 21 contrast images (i.e., 7 (sound location) x 3 (pre, postVA, postAV)). Moreover, we computed a contrast and associated t-image that compared all 21 sound conditions relative to fixation baseline (for identification of sound-responsive voxels).<br>For the multivariate decoding, we applied multivariate spatial noise normalization to the BOLD-response activation patterns using the noise covariance matrix obtained from the residuals of the GLM and the optimal shrinkage method and finally performed Euclidean normalization.<br>We performed the regional mean BOLD-response analysis using spatial and decisional uncertainty linear mixed effects models as follows. Regional mean BOLD-response: For each of the 2 (hemisphere: left, right) x 5 (ROI: HG, hA, IPS, IPL, FEF) regions we selected the 20 most reliably responsive voxels, i.e., with the greatest t-values for all unisensory sound conditions relative to fixation. For each of those 10 regions we extracted the BOLD-response magnitude for each of the 7 locations x 3 phases (pre-, postAV-, and postVA-adaptation) and formed the regional mean. Linear mixed effects modelling: To account for lateralization effects, we performed separate analyses for each region and hemisphere. Separately for each hemisphere we averaged activations of 360 simulated neurons the spatial (resp. decisional uncertainty) model to generate predictors for the regional mean BOLD-responses in each of the 21 conditions = 7 locations x 3 phases (pre-, postAV-, and postVA-adaptation).<br>We performed the multivariate decoding and representational similarity analyses as described in the Models & analysis section below.
Effect(s) tested	For the regional mean BOLD-response analysis, we generated seven linear mixed effects (LME) models that varied in their fixed effects predictors:<br>• Null LME: single intercept term.<br>• Spatial LME model (S): predictor from the spatial encoding model without recalibration and intercept term.<br>• Decisional uncertainty LME model (D): predictor from the decisional uncertainty model without recalibration and intercept term.<br>The remaining LME models included spatial, decisional uncertainty and intercept terms (i.e., 3 fixed effects regressors) and factorially manipulated whether the spatial and/or the decisional uncertainty predictor modelled recalibration:<br>• (S+D) Spatial without recalibration + decisional uncertainty without recalibration<br>• (SR+D) Spatial with recalibration + decisional uncertainty without recalibration,<br>• (S+DR) Spatial with recalibration + decisional uncertainty without recalibration<br>• (SR+DR) Spatial with recalibration + decisional uncertainty with recalibration<br>Subject level effects were included as random effects. For each of the 2 hemispheres x 5 ROIs we fitted these seven LME

models using maximum likelihood estimation and computed the Bayesian information criterion (BIC).

Specify type of analysis: ☐ Whole brain ☐ ROI-based ☒ Both

Anatomical location(s)

We defined five regions of interest (ROI, combined from two hemispheres) that have previously been implicated in auditory spatial processing based on neurophysiology and neuroimaging research. Heschl's gyrus (HG), higher auditory cortex (hA) and inferior parietal lobule (IPL) were defined using the following parcellations of the Destrieux atlas of Freesurfer 5.3.052: (i) HG: Heschl's gyrus and anterior transverse temporal gyrus; (ii) hA: higher auditory cortex, i.e., transverse temporal sulcus, planum temporale and posterior ramus of the lateral sulcus; (iii) IPL: inferior parietal lobule, i.e., supramarginal gyrus and inferior part of the postcentral sulcus. The intraparietal sulcus (IPS) and frontal eye field (FEF) were defined using the following group-level retinotopic probabilistic maps: (iv) IPS: IPS0, IPS1, IPS2, IPS3, IPS4, IPS5 and SPL1; (v) FEF: hFEF. All probabilistic maps were thresholded to a probability of 0.1 (i.e., probability that a vertex belongs to a particular ROI) and inverse normalized into each participant's native space.

Statistic type for inference
(See Eklund et al. 2016)

For regional mean BOLD-response analysis, see above. For multivariate decoding and representational similarity analyses, see below.

Correction

We used the Benjamini-Hochberg algorithm to correct for false discovery rate (FDR) across ROIs in the fMRI multivariate decodign analysis

Models & analysis

n/a	Involved in the study
☒	☐ Functional and/or effective connectivity
☒	☐ Graph analysis
☐	☒ Multivariate modeling or predictive analysis

Multivariate modeling and predictive analysis

At the first, within-subject level, we extracted the voxel response patterns in a particular ROI from the pre-whitened and normalized parameter estimate images pertaining to the magnitude of the BOLD-response for each condition and run. To avoid motor confounds, we used the parameter estimate images only from the 'non-response trials'. In a 4-fold stratified cross-validation procedure, we trained support vector regression models (C = 1, v = 0.5, LIBSVM 3.17) to learn the mapping from the condition-specific fMRI response patterns (i.e., examples) to external spatial locations (i.e., labels) using examples selectively from the unisensory auditory pre-adaptation runs of all but one fold. This learnt mapping was used to decode the spatial locations from the BOLD-response patterns of the remaining pre-adaptation fold and all postVA- and postAV-adaptation examples (acquired in separate runs).

We performed the multivariate decoding analysis using spatial encoding and recalibration indices and neurometric functions as follows. To determine whether a ROI encodes auditory spatial representations, we computed the Pearson correlation coefficients between the true and the decoded auditory locations for the pre-adaptation runs for each participant as a 'spatial encoding index'. To determine whether auditory spatial representations in a region of interest are recalibrated by misaligned visual signals, we binarized the predicted auditory locations into left vs. right predictions and computed the difference in the fraction of 'decoded right responses' between auditory postVA- and postAV-adaptation phases as 'recalibration index' (RI). At the second, between subject level, we entered the subject-specific Fisher z-transformed spatial encoding and recalibration indices into separate bootstrap-based one sample t-tests against zero at the group level.

In addition, we fitted cumulative Gaussians as 'neurometric functions' (NF) in each ROI to the percentage of 'decoded right' averaged across participants as a function of stimulus location. Consistent with our behavioural analysis we assessed whether AV- and VA-adaptation induced a shift in the decoded location of the unisensory auditory stimuli by comparing a 'static' model with a 'recalibration' model using the Akaike Information Criterion.

We performed representational similarity analysis using representational dissimilarity matrices, multidimensional scaling and pattern component analysis as follows. First, we generated 21 condition specific contrast images for the 7 auditory spatial locations x 3 (pre-, postVA-, and postAV-adaptation) by averaging parameter estimate images across fMRI runs for each participant. We then characterized the geometry of spatial representations using representational dissimilarity matrices (RDMs) based on the Mahalanobis distance for each participant and each ROI separately for pre-adaptation as well as postVA- and postAV-adaptation phases (see Supplementary methods). Second, using non-classical multidimensional scaling (MDS) with non-metric scaling, we projected the group level RDMs (i.e., averaged across participants) onto a one-dimensional space ('reflecting' spatial dimension along the azimuth). Third, to assess whether spatial and/or decisional uncertainty models can explain the fine-scale fMRI activity patterns we combined Pattern Component Modelling (PCM, https://github.com/jdiedrichsen/pcm_toolbox) and Bayesian model comparison. In particular, we generated second moment matrices ('pattern components') as predictors for PCM based on the activations of 360 simulated neurons from the spatial and decisional uncertainty models, respectively. We compared the following PCM models:

Null PCM: all conditions are independent (i.e., the 2nd moment matrix is the identity matrix).

Spatial PCM (S): activity patterns generated by the spatial model without recalibration.

Decisional uncertainty PCM (D): activity patterns generated by the decisional uncertainty model without recalibration.

Combined (spatial + decisional uncertainty) PCMs: activity patterns are a weighted linear combination of the patterns generated by the spatial and the decisional uncertainty model. We factorially manipulated whether the spatial and/or decisional uncertainty model accommodate audiovisual recalibration:

• (S+D) spatial component without recalibration and decisional uncertainty component without recalibration
• (SR+D) spatial component with recalibration and decisional uncertainty component without recalibration
• (S+DR) spatial component without recalibration and decisional uncertainty component with recalibration
• (SR+DR) spatial component with recalibration and decisional uncertainty component with recalibration

We estimated the parameters of the PCM models in a leave-one-subject-out cross-validation scheme. The marginal likelihood for each model and subject from was used as an approximation to the model evidence.
